# Transcutaneous fluorescence spectroscopy as a tool for non-invasive monitoring of gut function: first clinical experiences

**DOI:** 10.1038/s41598-020-73149-2

**Published:** 2020-09-30

**Authors:** James Maurice, Aaron M. Lett, Charlotte Skinner, Alexandra Lim, Matthew Richardson, Ajesh Painadath Thomas, Peter A. Summers, Khushi Vyas, Abdul Wadood Tadbier, Ramon Vilar, Marina K. Kuimova, Serge Miodragovic, Nikhil Vergis, Paul Kelly, Maria Francesca Cordeiro, Jonathan Hoare, Ara Darzi, Robert Goldin, Mark Thursz, Alex J. Thompson

**Affiliations:** 1 Department of Surgery & Cancer, St. Mary’s Hospital Campus, Imperial College London, London, W2 1NY UK; 2 Department of Metabolism, Digestion and Reproduction, Imperial College London, London, W2 1NY UK; 3 Imperial College Ophthalmology Research Group, Western Eye Hospital, Imperial College London, London, NW1 5QH UK; 4 Department of Chemistry, White City Campus, Imperial College London, London, W12 0BZ UK; 5 The Hamlyn Centre, Institute of Global Health Innovation, South Kensington, Imperial College London, London, SW7 2AZ UK; 6 Blizard Institute, Queen Mary University of London, London, E1 2AT UK; 7 Tropical Gastroenterology and Nutrition Group, University of Zambia School of Medicine, Lusaka, Zambia

**Keywords:** Gastrointestinal diseases, Fluorescence spectroscopy, Optical sensors

## Abstract

Gastro-intestinal function plays a vital role in conditions ranging from inflammatory bowel disease and HIV through to sepsis and malnutrition. However, the techniques that are currently used to assess gut function are either highly invasive or unreliable. Here we present an alternative, non-invasive sensing modality for assessment of gut function based on fluorescence spectroscopy. In this approach, patients receive an oral dose of a fluorescent contrast agent and a fibre-optic probe is used to make fluorescence measurements through the skin. This provides a readout of the degree to which fluorescent dyes have permeated from the gut into the blood stream. We present preliminary results from our first measurements in human volunteers demonstrating the potential of the technique for non-invasive monitoring of multiple aspects of gastro-intestinal health.

## Introduction

The function of the gut plays a pivotal role in many health disorders including coeliac disease, inflammatory bowel disease (IBD), HIV (human immunodeficiency virus), sepsis, chronic liver disease and environmental enteric dysfunction (EED). In particular, disruption of the gut barrier has important effects, leading to increased intestinal permeability and even translocation of gut bacteria and pathogen-associated molecular peptides into the systemic circulation causing inflammatory responses^1–4^.

The techniques currently used to assess intestinal function (particularly intestinal permeability) are either highly invasive (requiring endoscopic biopsy and histopathology, e.g.^3^.) or they are unreliable and difficult to perform in infants (e.g. Lactulose:Mannitol (L:M) or polyethylene glycol (PEG) permeability tests)^5^. Furthermore, the role of the gut in many of the above conditions is poorly understood. Together, this reveals a burgeoning need for new technologies that can enhance our understanding of gut function in the pathogenesis of disease and that can serve to provide earlier diagnosis and improved monitoring of the many illnesses in which it plays a role^6^.

Fluorescence spectroscopy presents opportunities for minimally invasive monitoring and diagnostics, and has been used in wide-ranging disease studies in both animals and humans. Indeed, fluorescence spectroscopy/imaging of orally introduced fluorescent contrast agents has been used in previous animal studies to quantify both intestinal permeability and intestinal closure^7–9^. In these studies, oral doses of fluorescent contrast agents (dyes) were first given to rats and/or pigs and concentrations were later measured (using fluorescence imaging/spectroscopy) in blood, tissue or urine samples to assess the degree to which the dyes had passed from the gut into the blood stream. These measurements were then used to provide a quantification of intestinal permeability or closure^7–9^. In the recent paper by Dorshow et al., the authors also reported detection of the fluorescent dyes through the skin on the ears of anaesthetised rats using a bifurcated optical fibre probe^9^, indicating the potential for non-invasive monitoring of gut permeability.

In this article, we report preliminary results from a first-in-human study assessing transcutaneous (through-the-skin) fluorescence spectroscopy as a tool for non-invasive monitoring of gut function. Our approach is analogous to the animal studies discussed above. Participants receive an oral dose of one or more fluorescent contrast agents and a wearable fibre-optic probe is used to detect the presence of those agents in the blood stream via transcutaneous fluorescence spectroscopy. By quantifying the resulting fluorescence signal in terms of parameters such as the rate of uptake, absolute intensity (with appropriate normalisation) and/or the ratio of intensities of different dyes, it may be possible to ascertain information that is useful in the diagnosis and monitoring of a range of gastro-intestinal (GI) disorders. For example, the rate of uptake of the fluorescent dyes (from the gut into the blood stream) may inform on the rate of gastric emptying (the rate at which the stomach empties following ingestion of a meal), which is impaired in conditions ranging from diabetic gastroparesis to stomach cancer. Similarly, the intensity of the fluorescence signal (and the ratio of intensities of different dyes) can be expected to vary in response to changes in gut permeability (which are known to occur in coeliac disease, IBD, HIV, EED and other conditions).

Physiological variations in permeability, gastric emptying rate and other elements of GI function are of course also expected within healthy populations (due to factors such as diet, alcohol intake, age, etc.). In addition, absolute fluorescence signals are likely to vary between individuals, for example because of differences in skin colour or body mass index (BMI). Nonetheless, the ability to non-invasively quantify parameters such as permeability and gastric emptying rate may present a series of opportunities to improve clinical decision making. For example, if the variability within healthy cohorts is small relative to the changes observed in disease states, it may be possible to screen for conditions in which altered permeability or gastric emptying rate are expected. Furthermore, longitudinal monitoring of responses to treatments/therapies (e.g. gluten free diets in coeliac disease, nutritional/hygienic interventions in malnutrition) may also be feasible (and it is noteworthy that longitudinal monitoring can circumvent problems caused by inter-patient variability, as changes in the detected signals/values are typically used for clinical decision making rather than absolute values). Thus, using transcutaneous spectroscopy to non-invasively monitor the uptake of orally ingested fluorescent contrast agents from the gut into the blood stream may provide a number of opportunities to improve clinical assessment of gut function.

To test the proposed technique, we developed and used a portable, dual-wavelength, fibre-optic fluorescence spectrometer to assess the feasibility of three fluorescent dyes for this purpose—fluorescein, indocyanine green (ICG) and fluorescein isothiocyanate conjugated dextran (FITC-Dextran). Preliminary results from measurements in 6 participants revealed that ICG was not detectable through the skin following oral ingestion, but that transcutaneous fluorescein fluorescence was readily detectable. Furthermore, FITC-Dextran could be detected in urine samples, indicating that higher concentrations/doses may be suitable for transcutaneous sensing. Overall, our results indicate the feasibility of non-invasive monitoring of gut function in the clinic using transcutaneous fluorescence spectroscopy of orally administered contrast agents, and suggest that the technique holds potential for sensing of permeability and other aspects of GI function.

## Results and discussion

### Portable fibre-optic fluorescence spectrometer

**Figure 1:**
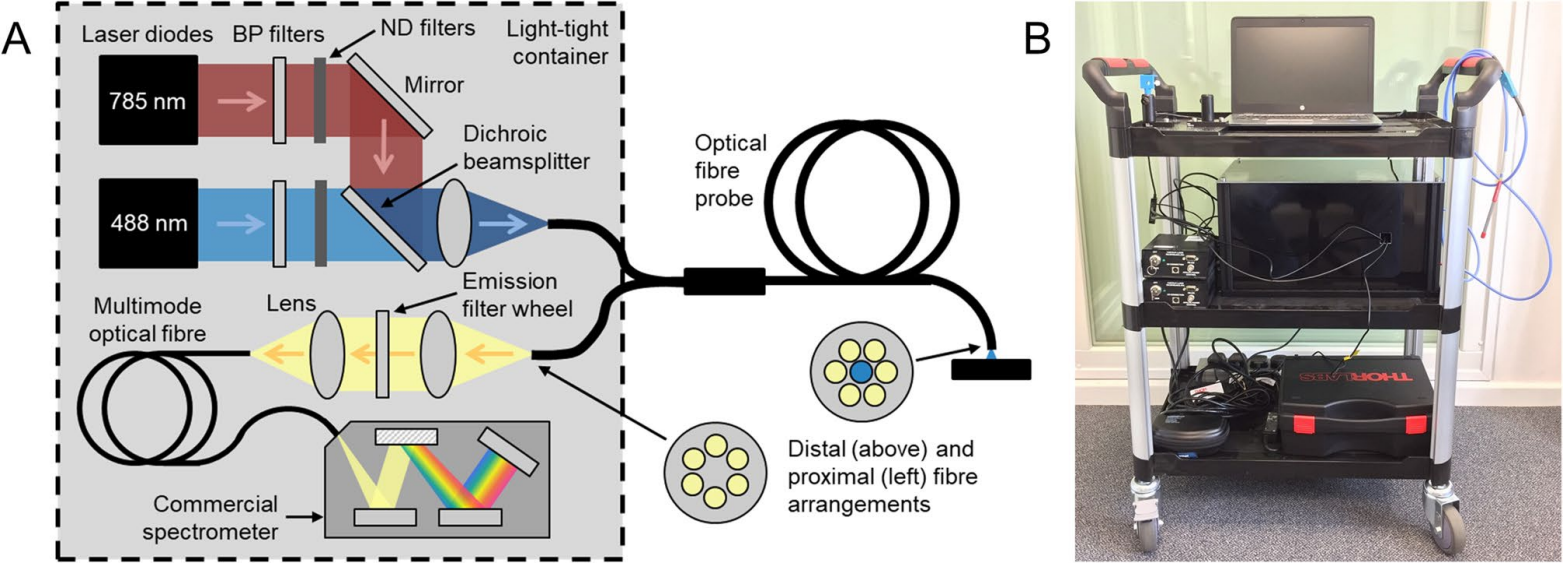
Portable fibre-optic fluorescence spectrometer. (**A**) Schematic diagram of the optical system. Insets show distal and proximal arrangements of optical fibres in the bifurcated fibre probe (excitation fibre—blue; collection fibres—yellow). ND—neutral density. (**B**) Photograph of the trolley-mounted spectrometer.

To facilitate testing of transcutaneous fluorescence spectroscopy as a tool for non-invasive monitoring of gut function, we developed a portable, fibre-optic fluorescence spectrometer that was suitable for deployment in clinical settings. This system is described in detail in the “Methods” section and in the clinical trial protocol for this study^10^. Briefly, however, the system consists of two laser sources (at 488 nm and 785 nm) for excitation of fluorescence, a commercial spectrometer, optical excitation/emission filters and an automated filter wheel (containing the emission filters) to ensure reliable detection of the fluorescence signals, and a bifurcated optical fibre probe to deliver light to (and collect light from) the participants’ skin (see Fig. 1A). Laser sources at 488 nm and 785 nm were chosen to permit excitation of fluorescence from fluorescein/FITC and ICG respectively (and from other dyes with comparable spectral properties). The optical system is contained within a light-tight aluminium box (height—25 cm, width—30 cm, length—45 cm) for the purposes of laser safety and is controlled by a laptop computer running LabVIEW software written in-house. The entire system is mounted on a wheeled trolley (Fig. 1B, dimensions: height—98 cm, width—63 cm, depth—42 cm) to allow use within clinics.

This portable spectrometer was used to make measurements of the transcutaneous fluorescence signals in study volunteers receiving either intravenous (IV) or oral doses of fluorescein, ICG and/or FITC-Dextran. These three fluorescent dyes were chosen as they have a range of molecular weights (the chemical structures of the dyes are shown in the Supplementary Information, Figure S1), and therefore potentially provide opportunities for assessment of intestinal permeability (e.g. through quantification of the relative uptake of dyes with different molecular weights). Transcutaneous fluorescence measurements were performed either by manually holding the fibre probe in contact with the participants’ skin or by securing it in place using 3D-printed mounts (Figure S2, Supplementary Information). Details of all clinical experiments are provided in the “Methods” and in Table 1. All measurements were performed according to the clinical study protocol^10^ and in accordance with Good Clinical Practice (GCP) guidelines and the World Medical Association’s Declaration of Helsinki. The experimental protocol received ethical approval following review through the UK Health Research Authority (HRA) and a local Research Ethics Committee (REC) (IRAS Project ID—242462, REC reference—18/LO/0714), and all participants gave informed consent prior to inclusion in the study.

**Table 1:** Summary of clinical experiments.

Participant number	Exp. number	Contrast agent(s)	Dose	Oral/IV	Other products consumed	Sensor position	Relevant figures	Additional Information
1	i	Fluorescein ICG	500 mg 25 mg	IV	N/A	Finger Arm Wrist	2	–
2	i	Fluorescein	500 mg	IV	N/A	Finger Arm Wrist	–	–
3	i	Fluorescein	500 mg	IV	N/A	Finger Arm Wrist	–	–
4	i	Fluorescein	2.5 g	Oral	100 ml water	Finger	3A-C	–
	ii	Fluorescein	330 mg	Oral	65 ml water	Finger	S3	Oral absorption experiment (no dye ingested)
	iii	Fluorescein	5 mg, then 25 mg	Oral	50 ml water (5 mg in 25 ml, then 25 mg in 25 ml)	Arm	3D	Limit of detection experiment
	iv	Fluorescein ICG	500 mg 250 mg	Oral	130 ml water, 60 g sugar	Arm	S4 S5	–
	v	Fluorescein	500 mg	Oral	100 ml water	Finger Arm Wrist	4	Confocal endo-microscopy experiment
	vi	Fluorescein	500 mg	Oral	1.5 g paracetamol, milkshake (see Table S1)	Finger	5A	Blood samples collected
5	i	Fluorescein	500 mg	Oral	100 ml water	Arm	–	–
	ii	Fluorescein ICG	500 mg 350 mg	Oral	140 ml water, 100 g sugar	Arm	S4 S5	–
	iii	FITC-Dextran	1 g	Oral	100 ml water, 60 g sugar	Finger	6 S5	Urine samples collected
6	i	Fluorescein	500 mg	Oral	100 ml water	Finger	5B	–
	ii	Fluorescein	500 mg	Oral	100 ml water, 60 g sugar	Finger	5B	–

*Exp* experiment, *IV* intravenous.

### Transcutaneous spectroscopy of IV contrast agents

**Figure 2:**
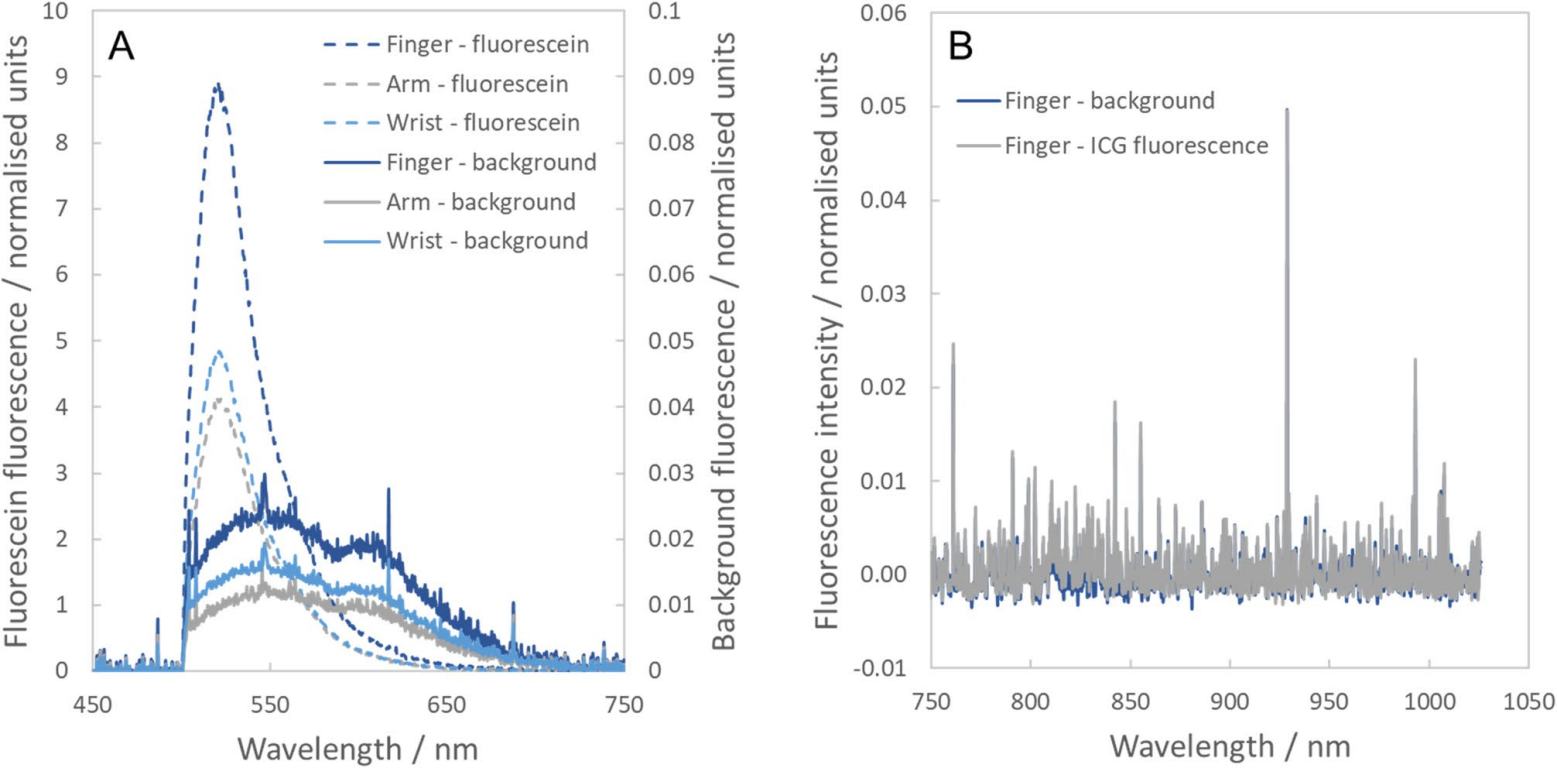
Transcutaneous spectroscopy of IV fluorescent contrast agents. (**A**) Fluorescence spectra observed in an ophthalmology patient who received an IV injection of fluorescein. Dotted lines represent measurements made after injection of fluorescein (left axis, 100 ms acquisition times) and solid lines represent background measurements recorded before injection of fluorescein (right axis, 5 s acquisition times). (**B**) Fluorescence spectra recorded in an ophthalmology patient who received an injection of ICG (blue—before injection; grey—after injection).

The portable spectrometer was used to assess the ability to detect transcutaneous fluorescence signals following IV injection of fluorescein and ICG. Three ophthalmology patients (participants 1–3) were recruited through Western Eye Hospital who were due to have injections of fluorescein and/or ICG as part of their clinical care. Two received injections of fluorescein alone and the third received injections of both fluorescein and ICG. For each patient, the fibre-optic spectrometer was used to measure the fluorescence signals at the skin in three different locations (tip of forefinger, back of forearm, and underside of wrist) before and after administration of the dyes. Clear fluorescein fluorescence was observed at all locations and in all patients, even with short acquisition times of 100 ms (Fig. 2A). Interestingly, the fluorescence intensity was higher at the fingertip than it was at either the forearm or the wrist. This is most likely due to the proximity of blood vessels to the surface of the skin.

While fluorescein was readily detectable, no ICG signal was observed above the background (Fig. 2B). This was true even when long acquisition times of up to 5 s were used. This result was attributed to the rapid clearance of ICG from the blood stream, which is known to occur at a rate of approximately 18–25% per minute^11^. While IV ICG is used to visualise blood vessels in the eye in retinal angiography examinations, its fluorescence signal is known to decrease very quickly after injection. In these experiments, the transcutaneous fluorescence measurements were performed several minutes after injection, after the patients had undergone retinal imaging (to avoid any unnecessary degradation of the patients’ clinical care). Thus, over this timescale it is likely that the ICG fluorescence will have reduced significantly, rendering the signals undetectable when measured through the skin. This suggests that ICG will not be suitable as a contrast agent for non-invasive monitoring of gut function following oral ingestion: as the dye would be expected to pass relatively slowly from the gut into the blood stream, the rapid clearance rate from the blood will be highly likely to make the fluorescence signal undetectable.

### Oral ingestion of fluorescein

#### Transcutaneous detection and temporal response

**Figure 3:**
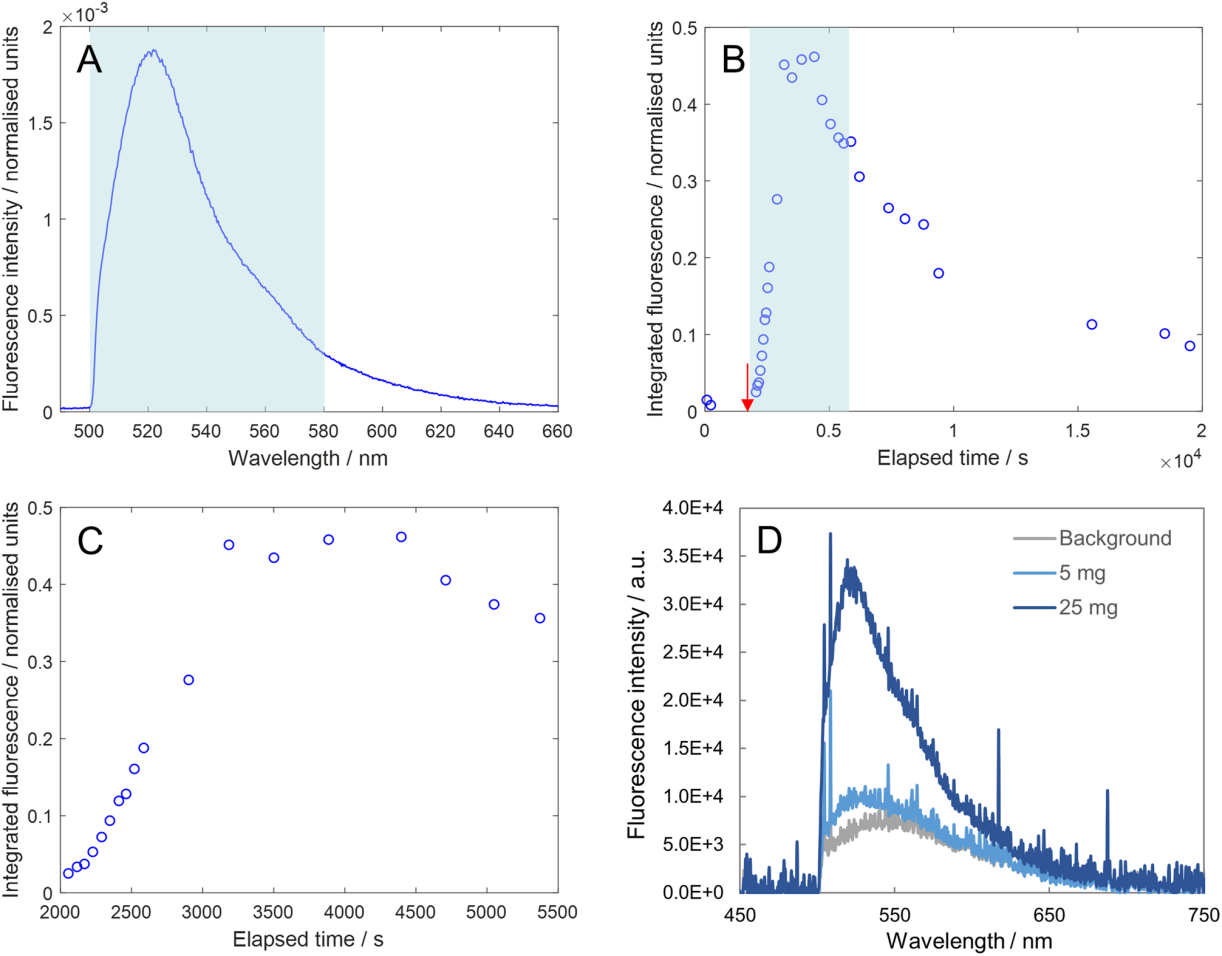
Transcutaneous spectroscopy of orally ingested fluorescein. (**A**) Example fluorescence spectrum recorded 16 min after ingestion of fluorescein (acquisition time = 250 ms). Blue shaded region indicates the wavelength range (500–580 nm) that was summed to calculate the normalised integrated fluorescence intensity (see details in “Methods”). (**B**) Normalised integrated fluorescence intensity as a function of time. The red arrow indicates the time at which the fluorescent dye was ingested by the participant (1920 s). (**C**) Close-up of the blue shaded region in (**B**) revealing an S-shaped uptake curve in the first hour after ingestion. (**D**) Spectra recorded in limit of detection experiment. Fluorescence from 5 mg of fluorescein was just detectable above the background (spectrum recorded 29 min after ingestion) while the signal from 25 mg of fluorescein was clearly observable (spectrum recorded 37 min after ingestion).

Following successful detection of IV fluorescein using transcutaneous spectroscopy, three healthy volunteers took part in a series of experiments investigating the ability to detect fluorescein after oral ingestion. In the first experiment, the first volunteer (participant 4) consumed 2.5 g fluorescein dissolved in 100 ml water and the fibre-optic fluorescence spectrometer was used to make measurements at the fingertip at 1–10 min intervals. After ingestion of the dye, clear transcutaneous fluorescence was detectable using the fibre-optic spectrometer with acquisition times as short as 100 ms (Fig. 3A).

To investigate how the fluorescence signal varied over time, an integrated fluorescence value was calculated at every time point and normalised according to both acquisition time and laser power (see “Methods”—“Calculation of normalised integrated fluorescence values”). As expected, the integrated fluorescence increased as a function of time after ingestion of the dye until a peak was reached and then slowly decreased back towards the background level (Fig. 3B). In this case the peak was observed approximately 2200 s after ingestion (peak at approx. 4100 s, dye ingested 1920 s (32 min) after initial background measurement). In addition, investigating the first hour after dye ingestion more closely revealed an S-shaped uptake curve (Fig. 3C), which provided a preliminary indication that the fluorescein was rapidly absorbed in the small intestine after it passed out of the stomach.

#### Negligible absorption through oral cavity

As fluorescence was observed quickly after ingestion (fluorescein fluorescence was observed above the background signal in the first measurement after ingestion, which was performed after 2 min), we also performed control experiments to confirm that no oral absorption of the dye had occurred. In this experiment, the same volunteer was asked to hold a fluorescein solution in their mouth for approximately 6 min and no fluorescence signal was observed (Figure S3, Supplementary Information), indicating that any oral uptake of the dye was negligible.

#### Limit of detection

As transcutaneous fluorescence was readily detectable at the dose used above, we also investigated the limit of detection. On a subsequent study day, participant 4 ingested a 5 mg dose of fluorescein (in 25 ml water) and transcutaneous fluorescence was measured at the arm for 31 min. Even at this very low dose (500 mg fluorescein is the typical dose used clinically), the fluorescence signal was just observable above the background (Fig. 3D). However, as the signal level was very low, participant 4 then ingested a further 25 mg fluorescein (in 25 ml water) and transcutaneous fluorescence spectra were recorded for a further 40 min. In this case, the fluorescence signal was clearly observable above the background, albeit with a relatively low signal-to-noise ratio (Fig. 3D). These results show that the limit of detection for fluorescein (using the current hardware) is below 25 mg. This indicates the clinical potential of this technique, as the fluorescein doses required for reliable detection are considerably lower than those used in other clinical procedures (e.g. up to 500 mg fluorescein for retinal angiography). In reality, it is likely that doses above 25 mg will be used in order to find the optimal trade-off between minimising the dose and maximising the signal-to-noise ratio. Indeed, in all further experiments presented here doses of 500 mg were used as this provided strong signal levels but also represented a dose that was used routinely in the clinic for other purposes.

#### Effect of sensing location

Following the successful proof-of-detection experiments above, we tested the effect that the location of the fibre-optic probe had on the data. Participants 4 and 5 took part in identical experiments to those presented in Fig. 3A–C but with the fibre-optic probe attached to the back of the forearm instead of the fingertip (and with a fluorescein dose of 500 mg). For both participants, an initial peak in the integrated fluorescence signal was reached at a broadly comparable time after dye ingestion to that observed in the first experiments (approx. 2200 s for participant 4, approx. 4300 s for participant 5—note that dye was ingested at 5 min (300 s) for both participants). Interestingly however, after that initial peak the fluorescence signal did not begin to decay back to the background level, but instead continued to increase before decaying at a much later time point than in the fingertip experiments (see Figure S4, Supplementary Information).

**Figure 4:**
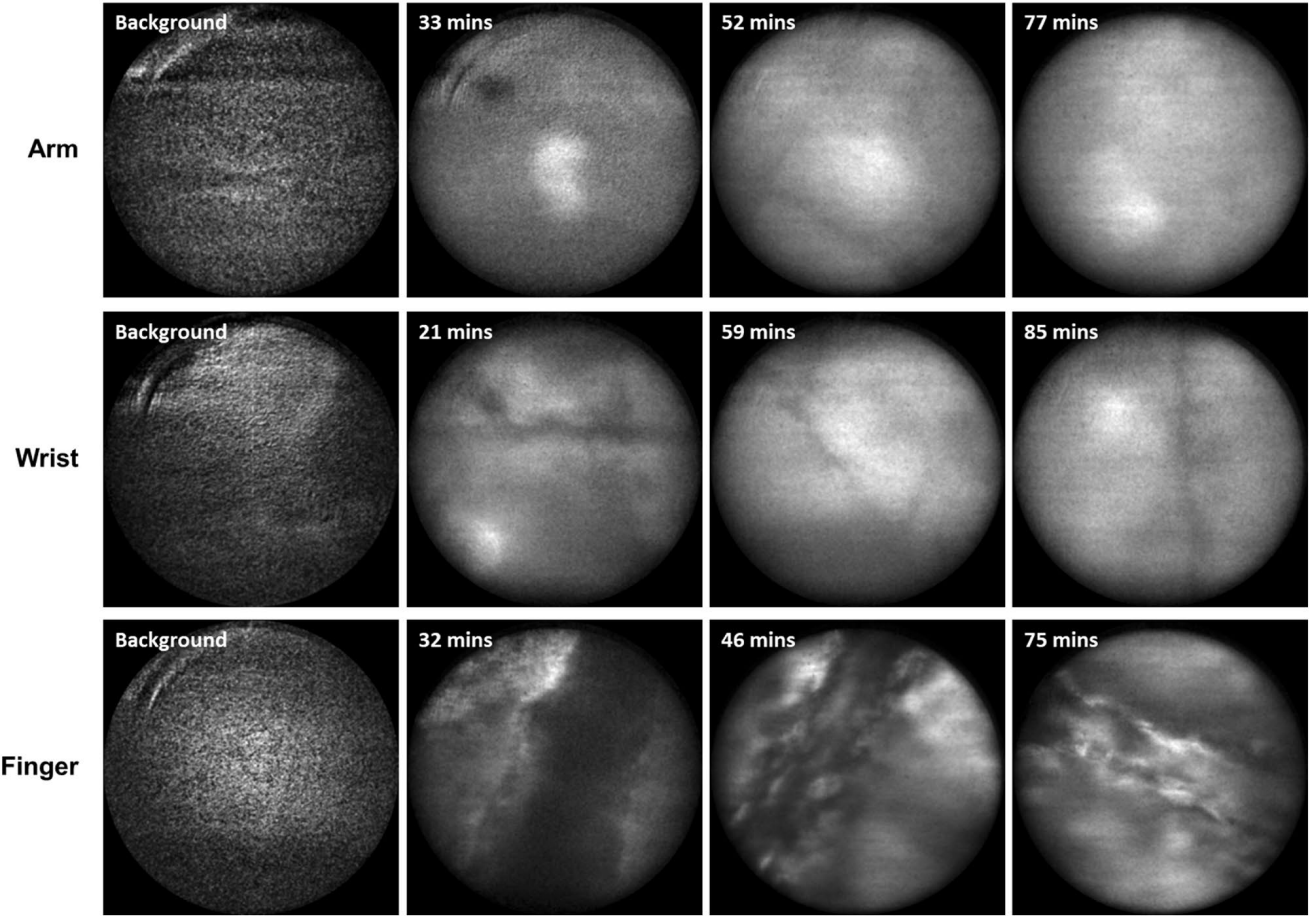
Confocal fluorescence endomicroscopy of skin following oral ingestion of fluorescein. Labels indicate times after ingestion. Diffuse fluorescence signals were observed at the arm and wrist, while more structured images were obtained at the fingertip. All images show a field of view with a 240 µm diameter.

To understand this effect, we performed confocal endomicroscopy to image the distribution of the dye at several body locations (fingertip, back of forearm and underside of wrist) after oral ingestion. Participant 4 again received a 500 mg dose of fluorescein (in 100 ml water) and a confocal fluorescence endomicroscope (developed in-house at Imperial College London^12^) was used to image the distribution of the dye at the finger, arm and wrist at intervals of approximately 5 min. As shown in Fig. 4, only a diffuse fluorescence signal was detected when recording images at the forearm and wrist, with no discernible structure observed. This was the case at all time points and the fluorescence intensity appeared to increase over time for the first 1–2 h (in qualitative agreement with the data shown in Figure S4, Supplementary Information). At the fingertip however, clear structure was observed with fluorescence appearing to emanate from cellular structures and/or blood vessels (Fig. 4).

Together, these results indicate that when the fibre-optic probe is placed at the fingertip it is most sensitive to the fluorescent signal from fluorescein in blood vessels. At the arm and wrist, however, the blood vessels are further below the surface of the skin. At early time points after dye ingestion, this means that the probe detects a diffuse signal that corresponds to multiply scattered light from dye in blood vessels. As the total signal intensity then increases over time (beyond the initial peak corresponding to the maximum concentration in the blood), this suggests that dye slowly leaks out of blood vessels and gets into the epidermis. At the arm and wrist (where blood vessels are further from the surface of the skin than they are at the fingertip), this leads to an increase in the fluorescence signal as the fluorescein gets closer to the sensing region of the probe. This signal remains for longer than that observed at the fingertip as the dye in the epidermis takes longer to be processed out of the body than the dye in the blood stream does. In addition, while this effect will also occur at the fingertip, the signal increase after the initial peak is less predominant as the proximity of the blood vessels means that the probe is preferentially sensitive to dye in the blood stream. Alternative probe designs could of course combat this effect (e.g. larger spacings between the source and detector fibres would allow for increased sensitivity to deeper blood vessels). Overall, however, this indicates that (of the three locations tested) the fingertip represents the optimal sensor position with the current hardware, as it will minimise the impact of dye leakage from blood vessels.

#### Small intestinal absorption

To provide physiologically relevant measurements of intestinal permeability, it will be necessary to assess the permeability in the small intestine (as this is the region in which increased permeability is known to occur in conditions such as coeliac disease, EED, etc.). Thus, to elucidate the region at which fluorescein is absorbed, we performed an experiment in which a healthy volunteer (participant 4) consumed both 500 mg fluorescein and 1.5 g paracetamol simultaneously (both were dissolved in a 400 ml milkshake—see Table S1, Supplementary Information). Transcutaneous fluorescence measurements were then made at 1 min intervals and blood samples were collected every 10–15 min to allow measurement of the blood paracetamol concentration. Paracetamol is known to be absorbed rapidly in the proximal small intestine^13–15^, so comparison of the time courses of fluorescein fluorescence and paracetamol concentration allowed us to assess whether fluorescein was also taken up in the small intestine.

**Figure 5:**
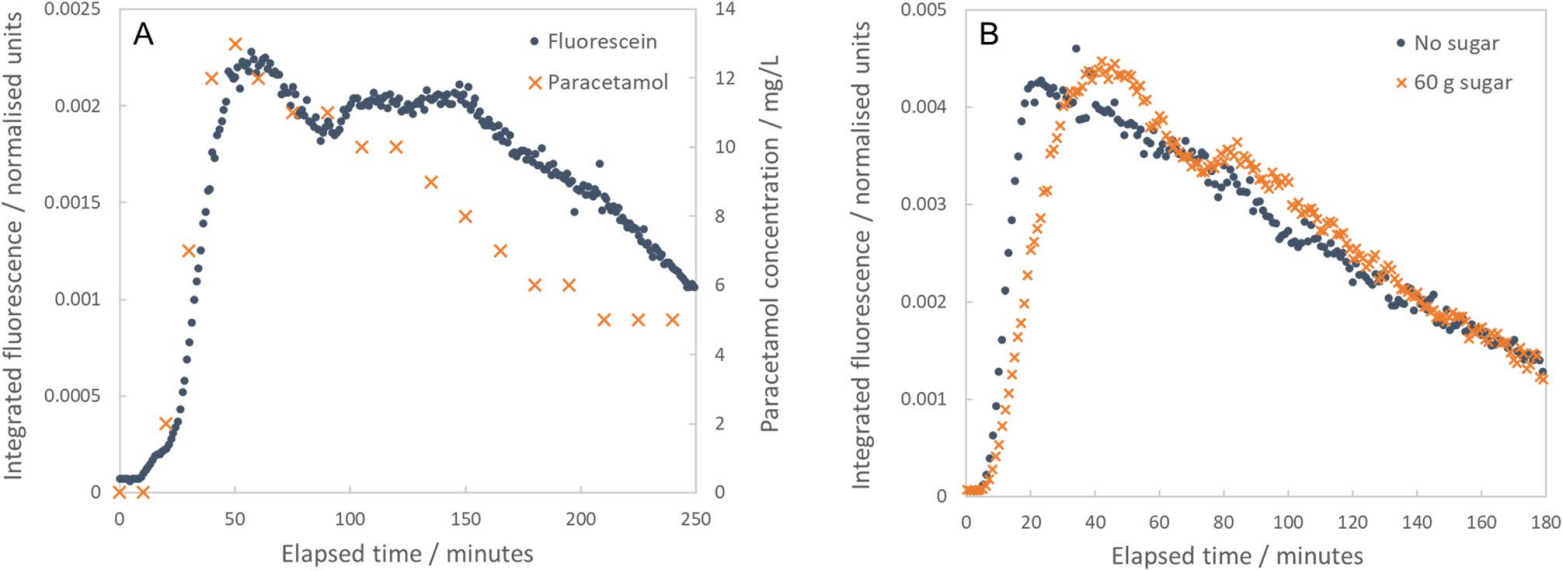
Comparison of transcutaneous fluorescence intensity and concentration of paracetamol in blood, and comparison of transcutaneous fluorescence signals with and without sugar. (**A**) Fluorescence intensity and paracetamol concentration plotted as functions of time. Measurements were made following oral ingestion of a milkshake containing both 500 mg fluorescein and 1.5 g paracetamol. Fluorescence intensity values (blue dots) are plotted on the left axis while paracetamol concentrations (orange crosses) are plotted on the right axis. The trends are in excellent agreement, particularly over the first 90 min after ingestion. (**B**) Two fluorescence intensity vs. time curves recorded in the same volunteer (participant 6) on separate days. In the first instance, only fluorescein was consumed (500 mg in 100 ml water—blue dots). In the second instance, fluorescein was consumed in combination with 60 g sugar (500 mg fluorescein, 60 g sugar, 100 ml water—orange crosses).

The fluorescein and paracetamol time courses were in excellent agreement, particularly over the first 90 min after ingestion (Fig. 5A). In combination with the results reported above, this indicates that fluorescein is taken up in the proximal small intestine, suggesting that it is a suitable fluorescent contrast agent for non-invasive permeability sensing via transcutaneous spectroscopy. In addition, as paracetamol concentrations in blood can be used to assess gastric emptying rates (as part of paracetamol absorption tests)^14–16^, this also suggests that transcutaneous spectroscopy of fluorescein may allow non-invasive assessment of gastric emptying rate (which is relevant to wide-ranging diseases including diabetic gastroparesis, dyspepsia, etc.). Indeed, a further indication of the potential for gastric emptying measurements is given by comparison of the fluorescein data presented in Figs. 3B,C and 5A. In Fig. 5A, the time taken to reach peak signal is approximately 3600 s (60 min) compared to 2200 s in Fig. 3B,C. Both experiments were performed in the same participant, but in Fig. 5A the dye was ingested as part of a milkshake (rather than in water as for Fig. 3B,C). As the milkshake would be expected to empty more slowly from the stomach than water would, this provides a secondary indication that transcutaneous spectroscopy of fluorescein may offer a non-invasive method for assessment of gastric emptying rates.

#### Fluorescein fluorescence intensity as a potential marker of intestinal permeability

It is also worth noting that the integrated fluorescence intensities observed at the first peak in the fluorescence vs. time curves varied across experiments performed in the same individual (participant 4). Following ingestion of a 2.5 g fluorescein dose in water, a peak value of approximately 0.46 was observed in the integrated normalised fluorescence data (Fig. 3B,C). Interestingly, a peak value of approximately 0.012 was observed after ingestion of 500 mg fluorescein in water (Figure S4A,C, Supplementary Information), while a 500 mg dose of fluorescein in a milkshake resulted in a value of 0.0023 (Fig. 5A). Hence, the peak fluorescence intensity appears to be dependent on the fluorescein dose and on the rate of uptake (a dose of 500 mg fluorescein in a milkshake corresponded to a lower peak intensity and a slower rate of uptake than the same dose in water). In turn, this suggests that—with the fluorescent signal appropriately normalised—transcutaneous spectroscopy of a single fluorescent contrast agent (in this case fluorescein) may provide a readout of gut permeability (i.e. as higher doses led to higher peak intensity values, this implies that permeation of larger quantities of dye would also correlate with higher intensities).

This is further supported by two repeat experiments performed in a final participant (participant 6, experiments i and ii) where transcutaneous fluorescence data was collected after oral ingestion of fluorescein. In the first case, participant 6 ingested a 500 mg dose of fluorescein (in 100 ml water). In the second experiment, the participant consumed a 500 mg dose of fluorescein combined with a 60 g dose of sugar (both dissolved in 100 ml water). The concentrated sugar solution is expected to have a hyperosmolar effect, driving a transient increase in intestinal permeability^17,18^. Thus, we expected to observe a higher peak fluorescence level in the second experiment. While the maximum values were in fact in close agreement (see Fig. 5B), the peak fluorescence value occurred considerably later in the second experiment (in which the sugar solution was consumed). Thus, following consumption of the sugar solution, the area under the curve (AUC)—calculated up until the time of peak fluorescence—was more than twice that observed when no sugar was ingested (Fig. 5B; AUC = 0.097 with sugar, AUC = 0.044 without sugar). Calculating the AUC up until the point of peak fluorescence provides an estimate of the total amount of fluorescein that has passed into the blood stream (albeit without any correction for the rate at which dye is eliminated from the body), with the time of the peak providing a correction for changes resulting from varying gastric emptying rate and/or varying absorption rate. Thus, the observation of a higher AUC value after ingestion of sugar provides further evidence that this approach may be able to differentiate states of varying permeability.

Interestingly, measurement of the fluorescence AUC value is analogous to measurement of total lactulose recovery in urine, which is often used in L:M tests to provide a quantification of permeability^19^. Importantly, however, total recovery of lactulose in urine (or any other urinary marker) is affected by the time at which urine is collected (as longer collection times will naturally lead to more of the marker accumulating in urine) and factors such as gastric emptying rate and absorption rate. It is for this reason that ratios (e.g. L:M) are typically used to assess permeability in urinary assays (ratiometric measurements provide a degree of correction for variations due to changes in gastric emptying rate, absorption rate, urinary collection time, etc.). Using transcutaneous fluorescence measurements can potentially solve this problem by providing direct measurements of dye permeation. As frequent measurements are made (e.g. once per minute as reported here), it is possible to accurately identify the time at which the peak concentration in the blood has been reached. Calculating the AUC up until that time point may then provide an accurate quantification of total dye recovery (analogous to total lactulose recovery). Thus, the AUC value can potentially act as a marker of intestinal permeability without the problems inherent in urinary assays, where the time of peak concentration in the blood is not known.

### Oral ingestion of ICG and FITC-Dextran

#### Testing of commercially available ICG and FITC-Dextran

We also investigated the potential of two other fluorescent dyes—ICG and FITC-Dextran—as contrast agents for non-invasive sensing of gut function via transcutaneous spectroscopy. Although ICG was not detectable following IV injection (see “Transcutaneous spectroscopy of IV contrast agents” section), we also tested whether transcutaneous fluorescence was observed following oral ingestion. Two participants (participants 4 and 5) received oral doses of ICG (with doses of 250 mg and 350 mg respectively). In both cases, no transcutaneous fluorescence was detected at any time after ingestion (Figure S5A, Supplementary Information).

**Figure 6:**
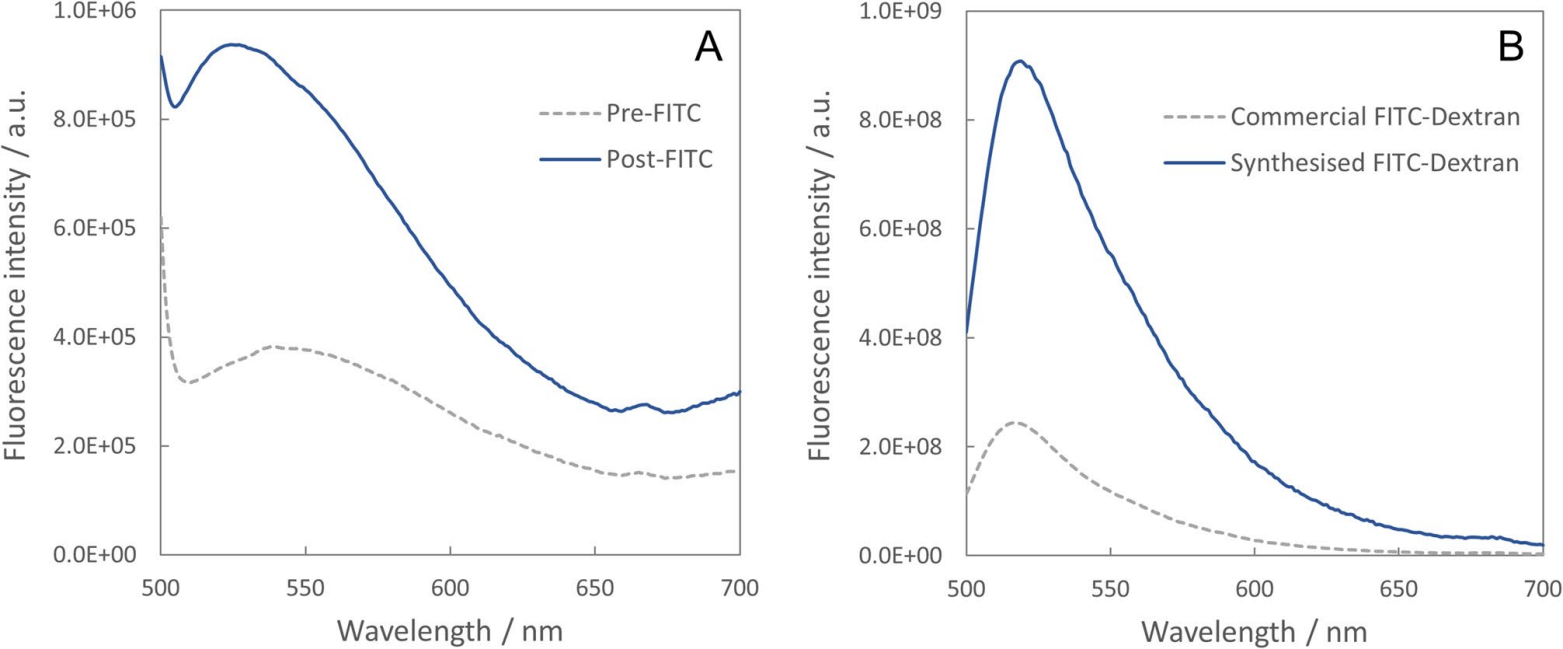
Fluorescence spectroscopy of FITC-Dextran. (**A**) Fluorescence spectra recorded in urine samples collected before (dotted grey line) and 2 h after (solid blue line) oral ingestion of 1 g FITC-Dextran (commercial product). (**B**) Fluorescence spectra observed in aqueous solutions of commercial and synthesised (2.5 eq) FITC-Dextrans demonstrating a 3.7 × increase in fluorescence intensity in the synthesised product. Spectra were normalised according to the concentration of each solution (6.9 × 10^–4^ mg/ml for the commercial product, 1.6 × 10^–4^ mg/ml for the synthesised product), with the volume used in each measurement kept constant (2 ml).

Similarly, in a separate experiment, participant 5 received a 1 g oral dose of FITC-Dextran and no transcutaneous fluorescence signal was observable above the background (Figure S5B, Supplementary Information). In this case, however, the participant also provided urine samples before and 2 h after ingestion of the FITC-Dextran. (No urine samples were collected in the ICG experiments as ICG is known to be extracted from the blood stream almost exclusively via hepatic clearance^11^, suggesting that little to no ICG would be detected in urine even if it did pass the gut barrier). Fluorescence spectra recorded from the urine samples revealed a higher fluorescence intensity in the post-FITC-Dextran sample than in the pre-FITC-Dextran sample (Fig. 6A). This indicated that FITC-Dextran had passed the gut barrier and was thus not detected simply because the dose was not high enough to facilitate detection through the skin (i.e. the transcutaneous fluorescence signal from FITC-Dextran was not high enough to be detected over the background fluorescence from skin). This implies that FITC-Dextran is potentially suitable as a contrast agent for transcutaneous monitoring of gut function, but that higher doses would be required to achieve this. (The 1 g dose used here was the highest allowable dose agreed in the current ethical approval).

It is worth noting here that in the above ICG and FITC-Dextran experiments, the participants consumed the fluorescent dyes in combination with a concentrated sugar solution (see “Methods” and Table 1). The sugar solutions were designed to have a hyperosmolar effect, acting to transiently increase gut permeability^17,18^, thereby increasing the likelihood that dyes would pass the gut barrier and be detected using transcutaneous spectroscopy. Thus, the lack of transcutaneous detection of both dyes indicates that higher doses/concentrations would be required even in patients with very permeable guts.

#### Testing of synthesised FITC-Dextran

As commercially available FITC-Dextran was not detectable through the skin, we investigated whether it was possible to synthesise FITC-Dextran with a higher FITC:dextran ratio and thus with a higher fluorescence intensity. The commercially available FITC-Dextran (purchased from Sigma-Aldrich) was quoted as having 0.002–0.02 mol of FITC per mole of glucose (dextran is a polymer of glucose). Based on a 1 g dose of FITC-Dextran and molecular weights of 389 g/mol and 4000 g/mol for FITC and dextran respectively, this corresponds to an effective FITC dose of between 4.8 mg and 48 mg (see calculation in Supplementary Information). As we previously showed that the limit of detection for fluorescein (which will pass the gut barrier to a much greater degree than FITC-Dextran) was approximately 25 mg, this explains why it was not possible to detect transcutaneous fluorescence from the orally ingested FITC-Dextran.

Thus, we synthesised FITC-Dextrans with higher molar concentrations of FITC relative to the commercial product (see “Methods” and Figures S6 and S7, Supplementary Information). Two batches of FITC-Dextran were prepared, using either 2.5 mol or 5 mol of FITC for every 1 mol of dextran (hereafter referred to as the 2.5 eq and 5 eq samples respectively). We characterised the spectral properties of the synthesised FITC-Dextrans. Absorption spectra showed the expected spectral shape for the 2.5 eq sample with peak absorption at approximately 494 nm (Figure S8A, Supplementary Information). For the 5 eq sample, however, a double-peaked absorption spectrum was observed (Figure S8B, Supplementary Information) that indicated aggregation of FITC (e.g. see^20^). Aggregation suggests that the concentration of FITC is too high as it typically leads to quenching of the fluorescence signal (e.g.^20^.). Nonetheless, we also recorded fluorescence spectra from both synthesised samples and from the commercial product for comparison. The 2.5 eq sample showed a fluorescence intensity that was approximately 3.7 times higher than that of the commercial product at the same concentration (Fig. 6B). Interestingly, the fluorescence intensity observed in the 5 eq sample was almost identical to that in the 2.5 eq sample (Figure S9, Supplementary Information), indicating that any increase in fluorescence intensity due to a higher concentration of FITC molecules was counteracted by quenching of the fluorescence due to FITC aggregation. Thus, the 2.5 eq FITC-Dextran was deemed to represent the optimal FITC:dextran ratio as it provided increased fluorescence intensity relative to the commercial product but without any fluorescence quenching effects.

To further confirm the above results, we calculated the FITC:dextran ratio in the 2.5 eq sample based on absorption spectra recorded at three different concentrations (Figure S8A, Supplementary Information) and the known extinction coefficient of FITC (77,000 M^−1^ cm^−1^ at 494 nm)^21^. This calculation yielded a FITC:dextran ratio of 0.74, which corresponds to approximately 0.02 mol of FITC per mole of glucose (see full details of calculation in Supplementary Information). This is up to 10 times higher than the molar ratio in the commercial product (0.002–0.02 mol of FITC per mole of glucose), in agreement with the 3.7-fold increase in fluorescence intensity observed above. This suggests that the synthesised 2.5 eq FITC-Dextran may be detectable via transcutaneous spectroscopy and future work will involve synthesising larger quantities to permit in vivo testing.

## Conclusions

We have presented a novel, non-invasive approach for the assessment of gut function (in particular gut permeability) based on transcutaneous fluorescence spectroscopy. The method involves participants/patients drinking an oral dose of one or more fluorescent contrast agents and a wearable probe being used to detect the presence of those agents in the blood. Importantly, measurements are made via fluorescence spectroscopy meaning that detection is non-invasive. Our preliminary results show that by quantifying the fluorescence signal (e.g. in terms of the absolute fluorescence intensity, the rate of uptake, the area under the fluorescence vs. time curve, etc.) it may be possible to assess permeability as well as other aspects of gastro-intestinal function (e.g. gastric emptying rate).

This approach has many potential advantages over other techniques used to assess gut function. As measurements are made through the skin without the need to collect urine samples, the technique is less invasive than other methods such as endoscopic biopsy and urinary permeability assays (e.g. L:M tests). In addition, data can be collected and analysed in an automated manner without the need to send samples to a laboratory for analysis, thereby facilitating faster reporting of results and lower costs. Finally, because data is collected directly as the fluorescent markers permeate from the gut into the blood stream (without the need for sample collection), this approach may provide increased reliability as well as more advanced data (i.e. fluorescence intensities can be measured every minute while urine is typically collected at one fixed time point of 2, 4 or 6 h). Thus, overall, transcutaneous spectroscopy has the potential to significantly improve upon current approaches.

We tested this technique in 6 volunteers (3 ophthalmology patients and 3 healthy volunteers) using three fluorescent dyes (fluorescein, ICG and FITC-Dextran). Our results show that fluorescein holds clear promise as a contrast agent for non-invasive sensing of gut function—it is detectable through the skin at low (clinically acceptable) doses, it is absorbed in the small intestine, and preliminary data indicates the potential to provide measurements of both gut permeability and gastric emptying rate. Neither ICG nor FITC-Dextran were detectable through the skin in the first instance. However, FITC-Dextran was detected in urine samples and we were also able to synthesise FITC-Dextrans with higher fluorescence intensities than commercially available products. This suggests that FITC-Dextran may also be suitable for use as a contrast agent for non-invasive sensing of gut function, but that higher FITC:dextran ratios and/or higher doses will be needed for this purpose.

In conclusion, preliminary clinical results have demonstrated that non-invasive monitoring of gut function is feasible using transcutaneous fluorescence spectroscopy of orally administered contrast agents. Importantly, this approach has the potential to improve on current techniques in terms of invasiveness, reliability, time taken to report results, and even cost. Future work will now involve more detailed clinical characterisation of the technique along with miniaturisation of the technology such that it is suitable for larger scale clinical deployment.

## Methods

### Portable fibre-optic fluorescence spectrometer

The fibre-optic spectrometer comprised two laser sources at 488 nm and 785 nm respectively (Stradus 488-25 and Stradus 785-80, Vortran Laser Technology, USA). Light from the two lasers was first passed through bandpass filters (LL01-488-12.5 (488 nm bandpass) and LL01-785-12.5 (785 nm bandpass), Semrock, USA) to clean up the emission and variable neutral density (ND) filters to limit the optical power to safe levels. The two laser beams were then combined using a dichroic beam splitter (FF506-DI03-25X36, Semrock, USA) and coupled into the excitation channel (central fibre) of a bifurcated fibre-optic probe (QR200-7-VIS-NIR, Ocean Optics, The Netherlands). This delivered the excitation light to the measurement site and the fluorescence was then collected by the detection channel (6 outer fibres) of the probe and routed back to the optical system. The output from the detection fibres was collimated and passed through a motorised emission filter wheel (FW102C, Thorlabs, USA), which contained two long pass filters to reject backscattered excitation light (FF01-496/LP-25 (496 nm long pass) and BLP01-785R-25 (785 nm long pass), Semrock, USA). The fluorescence was then coupled into a multimode optical fibre (P600-1-VIS-NIR, Ocean Optics, The Netherlands) that routed the light to a spectrometer for detection (FLAME-S-VIS-NIR-ES, Ocean Optics, The Netherlands). The layout of the optical system (including the arrangements of excitation and detection fibres in the bifurcated fibre probe) is shown in Fig. 1A. All optical components were enclosed within an anodized aluminium box and the system was mounted on a wheeled trolley to allow transportation to and within clinics (Fig. 1B).

The optical powers at the distal tip of the fibre probe were limited to 63 µW at 488 nm and 93 µW at 785 nm. This was achieved using a combination of the variable ND filters and the laser control software (Stradus GUI Software, Vortran Laser Technology, USA) and ensured that the laser exposures were below the maximum permissible exposures for the skin^22–24^. At these power levels, the distal laser output was also below the maximum permissible exposure for the eye with the tip of the probe held at a distance of > 10 cm (assuming a blink reflex of 0.5 s)^22–24^, meaning that the system could be safely used within a clinical environment.

The system was controlled using LabVIEW software written in-house. This allowed data to be collected in a manual or automated manner. For manual measurements, spectra were recorded at chosen time points using the desired emission filter and acquisition settings (e.g. integration time, number of averages, etc.). For automated measurements, spectra were recorded at regular time intervals (typically once every minute) with integration times determined by the software to ensure that sufficient signal-to-noise ratios were obtained regardless of the absolute signal levels. Specifically, for each time point, repeated spectra were collected with 500 ms integration times and these were summed until a signal threshold was reached. For each fluorescence spectrum, the backscattered laser signal was also recorded (1 ms integration time, 50 averages) by setting the emission filter wheel to an empty port (Figure S10, Supplementary Information). The laser spectra were used to normalise the corresponding fluorescence spectra to account for variations in laser power.

For all clinical measurements, the fibre-optic probe was held in gentle contact with the participants’ skin. This was either achieved manually or using one of two 3D-printed mounts, which used Velcro straps to secure the probe at the fingertip, arm or wrist (Figure S2, Supplementary Information). The 3D-printed mounts were designed to secure the probe perpendicular to the participants’ skin, which helped to minimise signal variations between measurements caused by changes to the inclination of the probe.

### Clinical trial measurements

Clinical measurements were performed at Western Eye Hospital (ophthalmology patients, participants 1–3) and St. Mary’s Hospital (healthy volunteers, participants 4–6) in London, UK. Participants were recruited and consented according to the clinical study protocol^10^, which received ethical approval through the UK HRA and a local REC (IRAS Project ID—242462, REC reference—18/LO/0714). The study was conducted in accordance with GCP guidelines and the World Medical Association’s Declaration of Helsinki, and all participants gave informed consent prior to inclusion in the study. A summary of all clinical experiments is shown in Table 1, which provides details of the contrast agents and doses used, the measurement locations, and the doses of any further components included in the ingested solutions (e.g. paracetamol, sugar, milkshake, water, etc.). Details of the milkshake given to participant 4 (experiment vi) are shown in Table S1 (Supplementary Information). The form, volume and macronutrient composition of the milkshake were designed to conform to the guidelines proposed by Willems et al*.*^16^ for standardised paracetamol absorption tests for gastric emptying rate. Fluorescent dyes were purchased through Imperial College Healthcare NHS Trust Pharmacy (Fluorescein—Anatera, Alcon, Novartis Pharmaceuticals, UK; ICG—Verdye, Diagnostic Green GmbH, Germany) and Sigma-Aldrich (FITC-Dextran—FD4, Sigma-Aldrich, USA). Fluorescein was provided in aqueous solution (500 mg dissolved in 5 ml water) while ICG and FITC-Dextran were provided as powders that were dissolved in water at the time of the experiments. Prior to ingestion, FITC-Dextran solutions were sterilised by passing them through 0.2 µm syringe-driven filters (Sterile Syringe Filter with 0.2 µm Polyethersulfone Membrane, VWR International, USA).

For ophthalmology patients (participants 1–3), measurements were made at the fingertip, arm and wrist (via manual placement of the fibre probe) for approximately 5 min following injection of fluorescein/ICG (with measurements beginning within 5–10 min of injection). For healthy volunteers (participants 4–6), the fluorescent contrast agents were delivered orally and measurements were made either using manual probe placement or with the probe secured in contact with the skin using one of the 3D-printed mounts (Figure S2, Supplementary Information). Spectra were then recorded for up to 4 h to allow investigation of the kinetics of the observed fluorescence signals.

In one case (participant 5, experiment iii), urine samples were collected before and 2 h after ingestion of the fluorescent dye (FITC-Dextran) to permit further assessment of whether the dye had passed the gut barrier. Similarly, blood samples were collected at 10–15 min intervals in one experiment (participant 4, experiment vi) to allow correlation of the fluorescence signal with serum concentrations of paracetamol (which was administered orally at the same time as the fluorescent dye).

Finally, in one experiment (participant 4, experiment v), a confocal fluorescence endomicroscope^12^ was used to image the distribution of fluorescein beneath the skin following oral ingestion. Further details of this experiment are provided below.

### Calculation of normalised integrated fluorescence values

For experiments involving oral ingestion of contrast agents, we calculated the total (integrated) fluorescence intensity at each time point to allow investigation of trends with respect to time. To achieve this, the background signal was first subtracted from the fluorescence spectrum for each time point. This was calculated as the average intensity over the wavelength range 350–450 nm (over which no signal was observed). The background-subtracted spectra were then summed over the wavelength range containing the spectral peak of the fluorescence signal (500–580 nm for fluorescein, 800–880 nm for ICG). This integrated fluorescence value was then normalised according to both integration time and laser power (by dividing by the product of the two). Laser power values were calculated based on the laser spectra (which were recorded immediately after each fluorescence spectrum—see Figure S10, Supplementary Information). To obtain the laser power values, the laser spectra were background subtracted as described above and were then summed over the ranges 485–492 nm and 778–785 nm for the 488 nm and 785 nm laser respectively. These summed values were then normalised according to the integration time used to collect the laser spectra (typically 1 ms). Thus, the normalised integrated fluorescence intensity values for fluorescein were calculated according to Eq. (1).

1 $${I}_{int}=\frac{\left(\sum_{\lambda =500nm}^{\lambda =580nm}\left(I(\lambda )-{B}_{F}\right)\right)\times {t}_{L}}{\left(\sum_{\lambda =485nm}^{\lambda =492nm}\left(L(\lambda )-{B}_{L}\right)\right)\times {t}_{F}}$$

Here, *I*_*int*_ is the integrated, normalised fluorescence intensity, *λ* is the wavelength, *t*_*F*_* and t*_*L*_ are the integration times for the fluorescence and laser spectra respectively, *I*(*λ*) is the fluorescence spectrum, *L*(*λ*) is the laser spectrum, and *B*_*F*_ and *B*_*L*_ represent the background values for the fluorescence and laser spectra respectively.

Importantly, this normalisation procedure involved measurement of the laser power at the measurement site (i.e. the skin) and not directly at the laser output. As such, it allowed numerous sources of signal variation to be accounted for including movement of the probe, changes in the pressure with which the probe was held in contact with the skin, fluctuations in laser power, and even variations in skin tone (i.e. across different individuals or different body locations). Combined with the use of 3D-printed mounts that acted to secure the tip of the probe perpendicular to the skin, this meant that the normalised fluorescence intensities could be accurately compared across different time points, body locations and even participants.

While this procedure was able to correct for the above variations, the data collection was still susceptible to changes on short timescales that occurred between collection of an individual fluorescence spectrum and the equivalent laser spectrum that was used for normalisation. For example, if the inclination of the probe changed between collection of a fluorescence spectrum and collection of the equivalent laser spectrum, this led to anomalous normalisation. However, this did not present a significant problem as spectral acquisition times were typically on the order of 0.5–5 s and variations on this timescale were rare.

### Confocal endomicroscopy

For the confocal endomicroscopy experiment presented in Fig. 4 (participant 4, experiment v), we used a custom-built line-scanning confocal endomicroscope (excitation wavelength—488 nm; detection wavelength range—517–547 nm) that was developed in-house at Imperial College^12^. This system was used to acquire images at 60 frames/s with an exposure time of 30 ms per line, a line width of 4 µm, and a detector gain of 18 (voltage gain). Images were recorded by holding the endomicroscope’s fibre-optic imaging probe in gentle contact with the skin at the fingertip, forearm and underside of the wrist. Data collection began immediately after the participant consumed a 500 mg dose of fluorescein (in 100 ml water) and images were collected at approximately 5 min intervals for 3.5 h. Background images were also recorded at each site prior to consumption of the dye.

### Synthesis of FITC-Dextran

Syntheses of FITC-Dextran conjugates were performed with two different ratios—2.5 equivalent moles (2.5 eq) and 5.0 equivalent moles (5 eq)—of FITC with respect to the dextran (6 kDa) concentration. Details of the synthetic procedure used with 2.5 eq FITC are given below.

In a 50 ml round bottom flask, FITC (81 mg, 0.208 mmol) and N,N′-carbonyldiimidazole (34 mg, 0.208 mmol) were dissolved in dry tetrahydrofuran (12 ml) and stirred at room temperature for 12 h. Dextran (6 kDa, 500 mg, 0.083 mmol) and triethylamine (30 µl, 0.208 mmol) were added, and the reaction mixture was stirred for another 24 h. The FITC-labelled dextran was then purified by dialysis against distilled water using a modified cellulose membrane (MWCO 2000) for 36 h. The water was changed at regular intervals to remove free FITC and other unwanted reagents. The solution was lyophilized (freeze dried) to yield the FITC-Dextran conjugate as an orange powder.

We performed liquid chromatography–mass spectrometry (LC–MS) to characterise the FITC-Dextran. Using a large (C4) LC column, this revealed the presence of mass components at approximately 6060 g/mol, corresponding to the labelled 6 kDa dextran (see Figure S7, Supplementary Information). Measurements using a small (C18) LC column then indicated that there was no free FITC present in the synthesised compound (i.e. no components were observed with a molecular weight of 389 g/mol). We also washed the FITC-Dextran in acetone to test for the presence of free FITC (FITC is soluble in acetone at 1 mg/ml), however, no fluorescence was observed in the wash solution. Together, these results indicated that the synthesised compound corresponded to FITC-Dextran with no free FITC present.

### Spectral characterisation of fluorescent dyes and urine samples

Spectral characterisation of the synthesised FITC-Dextrans and the urine samples collected from participant 5 (experiment iii) was performed using a UV–VIS absorption spectrometer (Cary 8454 UV–Visible Spectrophotometer, Agilent, USA) and a spectrofluorometer (FluoroMax-4, Horiba, Japan). For fluorescence measurements, an excitation wavelength of 488 nm was used along with a detection range of 500–700 nm (recorded in 1 nm increments). Entrance and exit slit sizes were set to 5 nm and an integration time (scan speed) of 0.1 s/nm was used. For UV–VIS absorption measurements, spectra were collected over the range 190–1100 nm with an integration time of 0.5 s.

## Supplementary information

Supplementary Information.
