# Supplementary material for: Transcutaneous fluorescence spectroscopy as a tool for non-invasive monitoring of gut function: first clinical experiences
Source: Sci Rep. 2020 Sep 30;10:16169. doi: 10.1038/s41598-020-73149-2 (PMC7527451; doi:10.1038/s41598-020-73149-2)
Supplement: Supplementary file 1 — Supplementary Information. [file 41598_2020_73149_MOESM1_ESM.pdf]

SUPPLEMENTARY INFORMATION

Transcutaneous fluorescence spectroscopy as a tool for non-invasive monitoring of gut function: first clinical experiences

James Maurice[1], Aaron M. Lett[2], Charlotte Skinner[2], Alexandra Lim[2], Matthew Richardson[3], Ajesh Painadath Thomas[4], Peter A. Summers[4], Khushi Vyas[5], Abdul Wadood Tadbier[1, 5], Ramon Vilar[4], Marina K. Kuimova[4], Serge Miodragovic[3], Nikhil Vergis[2], Paul Kelly[6, 7], Maria Francesca Cordeiro[3], Jonathan Hoare[1], Ara Darzi[1, 5], Robert Goldin[2], Mark Thursz[2], Alex J. Thompson[1, 5]*

[1]Department of Surgery & Cancer, St. Mary's Hospital Campus, Imperial College London, W2 1NY, UK
[2]Department of Metabolism, Digestion and Reproduction, Imperial College London, W2 1NY, UK
[3]Imperial College Ophthalmology Research Group, Western Eye Hospital, Imperial College London, NW1 5QH, UK
[4]Department of Chemistry, White City Campus, Imperial College London, W12 0BZ, UK
[5]The Hamlyn Centre, Institute of Global Health Innovation, South Kensington, Imperial College London, SW7 2AZ, UK
[6]Blizard Institute, Queen Mary University of London, E1 2AT, UK
[7]Tropical Gastroenterology and Nutrition Group, University of Zambia School of Medicine, Lusaka, Zambia

*Corresponding author: alex.thompson08@imperial.ac.uk

This PDF file includes:

Supplementary Figures S1-S10

Supplementary Table S1

Calculations of FITC masses/concentrations in commercial and synthesised FITC-Dextran

Supplementary Figures

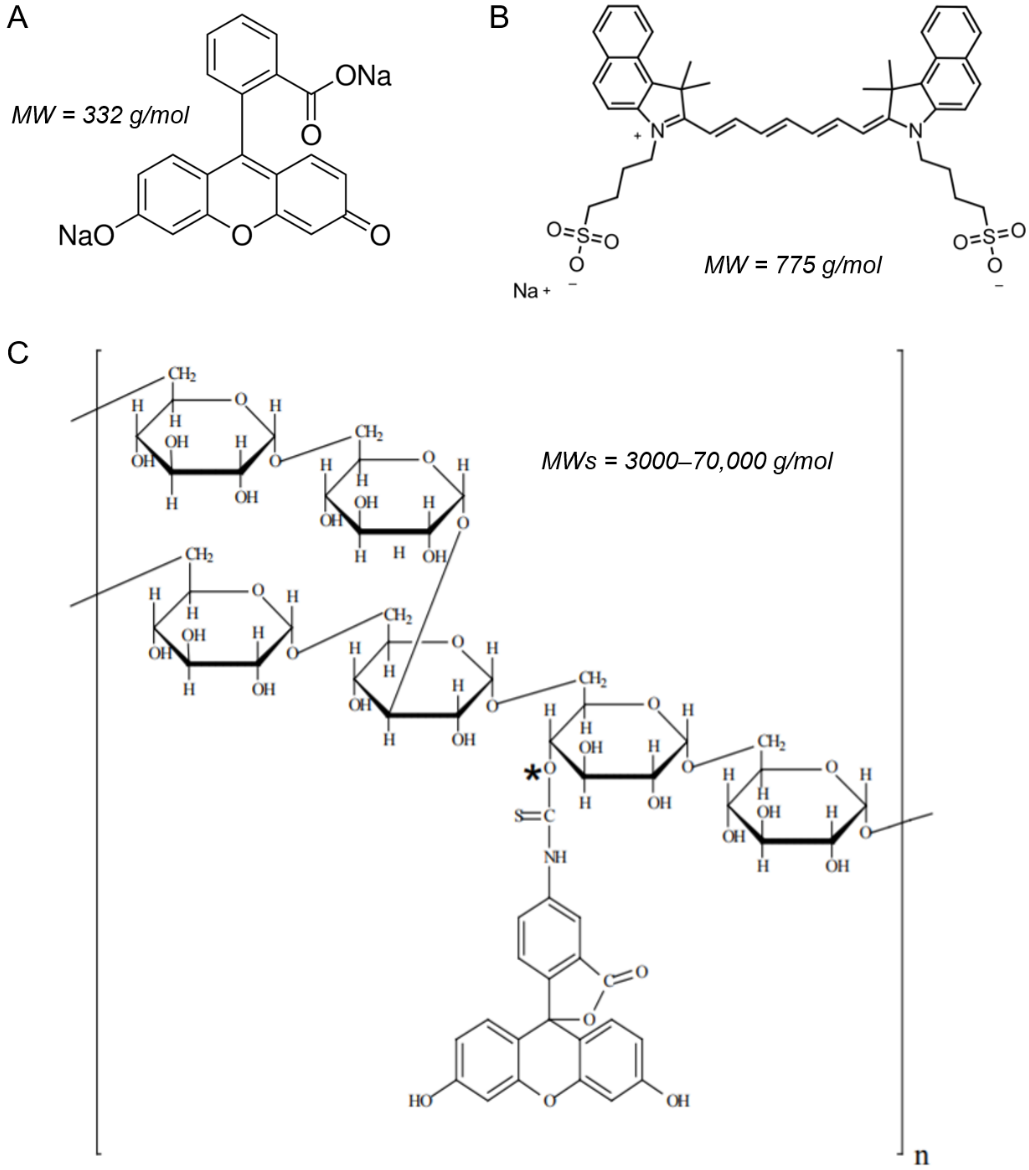

Figure S1. Chemical structures of the three fluorescent contrast agents investigated in this study. **(A)** Fluorescein, **(B)** Indocyanine Green (ICG), **(C)** FITC-Dextran. Molecular weights (MWs) are shown as insets.

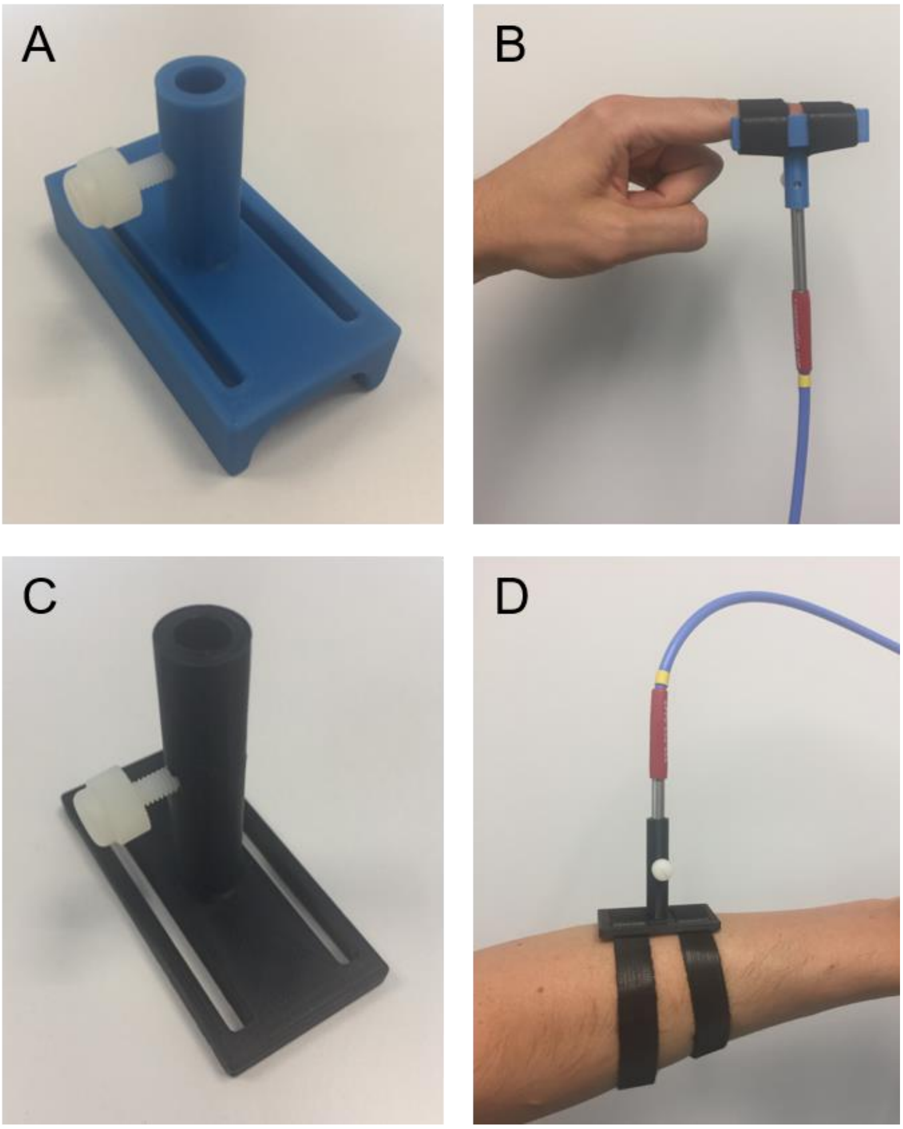

Figure S2. 3D-printed mounts to secure optical fibre probe at participants' skin. **(A, B)** Mount used to position probe in contact with fingertip. **(C, D)** Mount used to position probe in contact with arm or wrist.

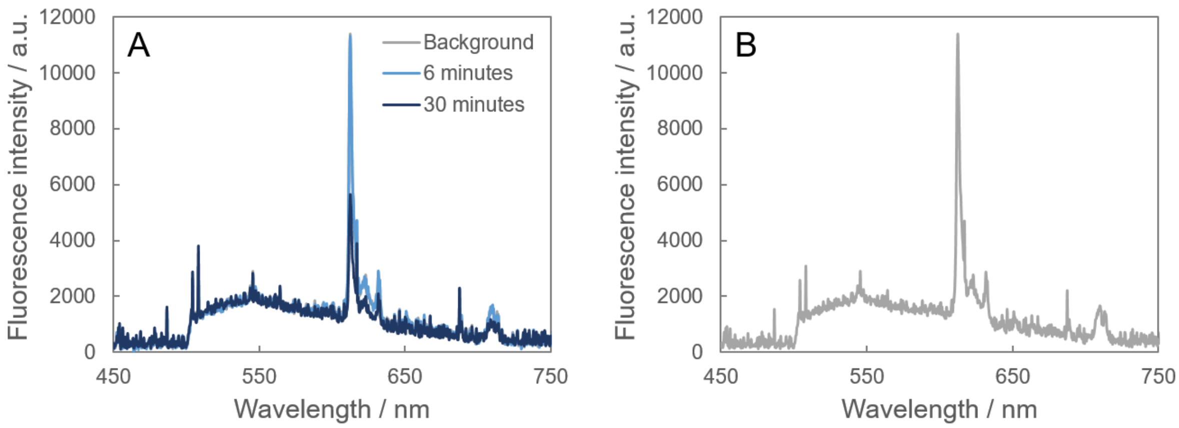

Figure S3. Results of oral absorption control experiment (participant 4, experiment ii). **(A)** Spectra recorded before (background) and both 6 minutes and 30 minutes after the subject began to hold a fluorescein solution in their mouth. The subject held the solution in their mouth for a total of 6 minutes before spitting. **(B)** Background spectrum only. No differences are observed between the three spectra indicating that no oral absorption of fluorescein occurred.

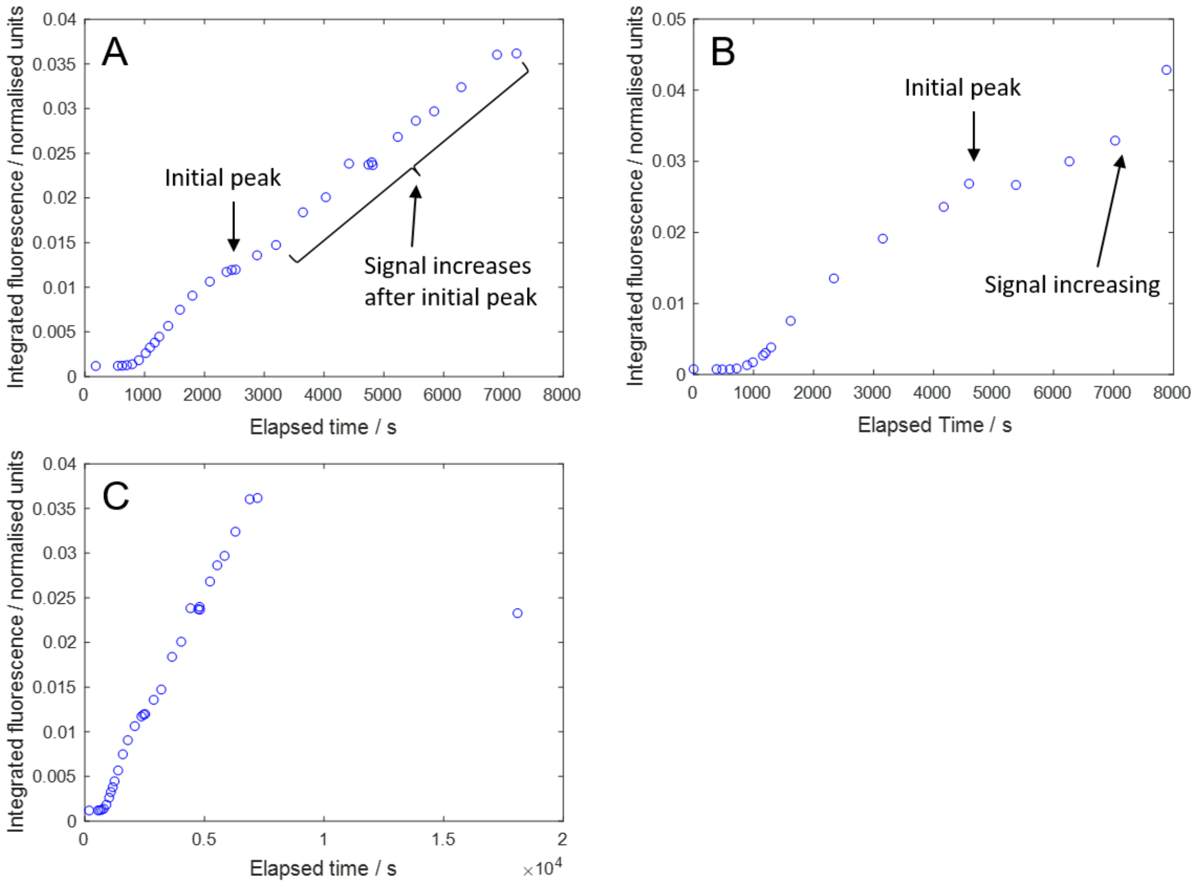

Figure S4. Transcutaneous fluorescence spectroscopy of orally ingested fluorescein with measurements made at the forearm. **(A)** Participant 4 (experiment iv) – first 8000 seconds. **(B)** Participant 5 (experiment ii) – full experiment. In both participants, the signal continues to increase after an initial peak. **(C)** Participant 4 (experiment iv) – full experiment, demonstrating that the signal level decreases at later time points.

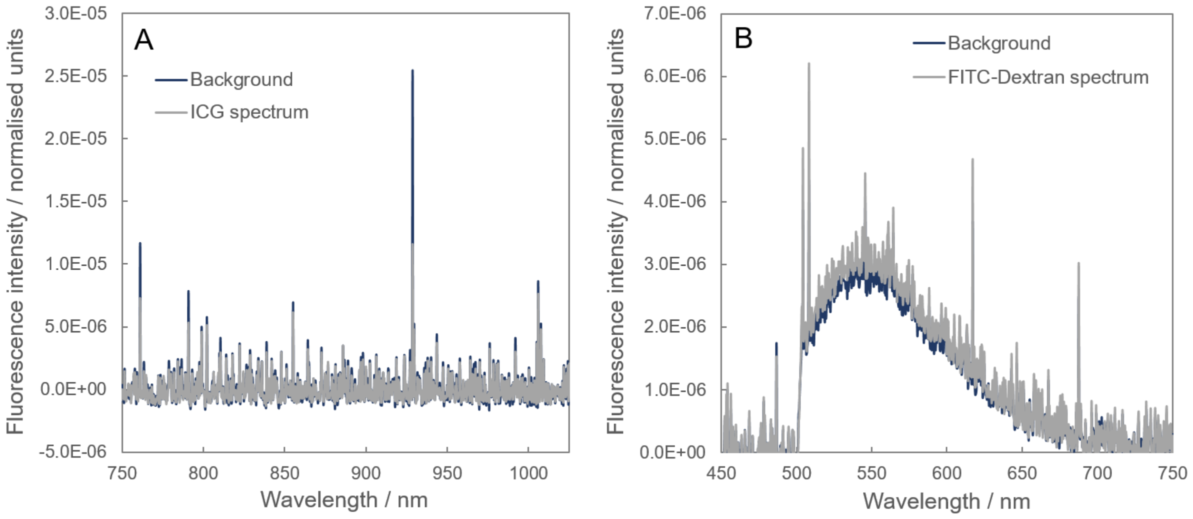

Figure S5. Transcutaneous fluorescence spectroscopy of orally ingested ICG **(A)** and FITC-Dextran **(B)**. In both cases, no signal is observable above the background.

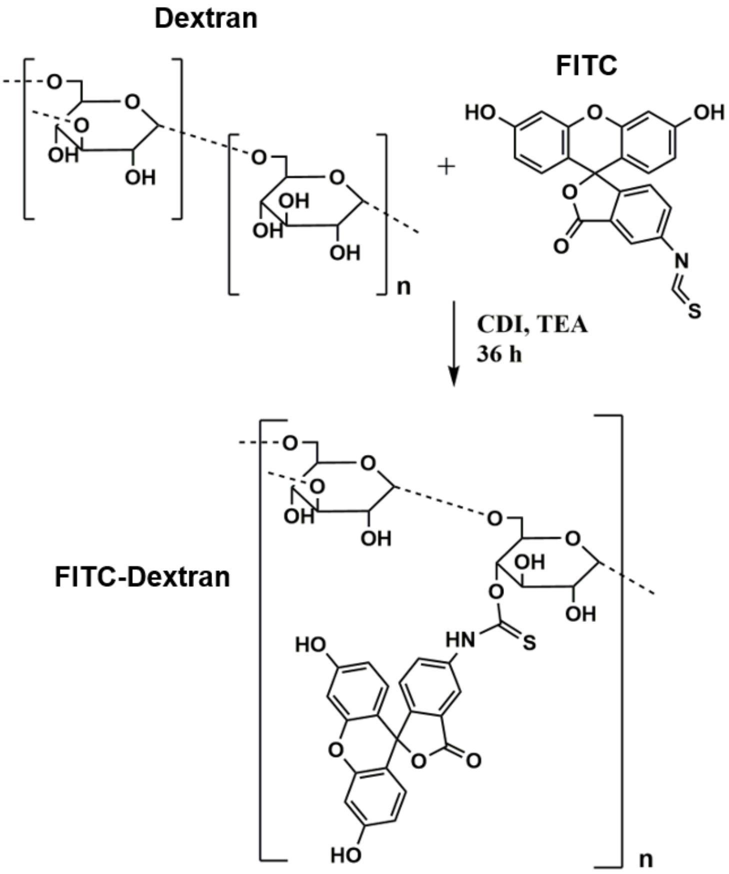

Figure S6. Synthesis scheme for fabrication of FITC-Dextrans with increased molar concentrations of FITC. CDI - N,N'-carbonyldiimidazole; TEA – triethylamine.

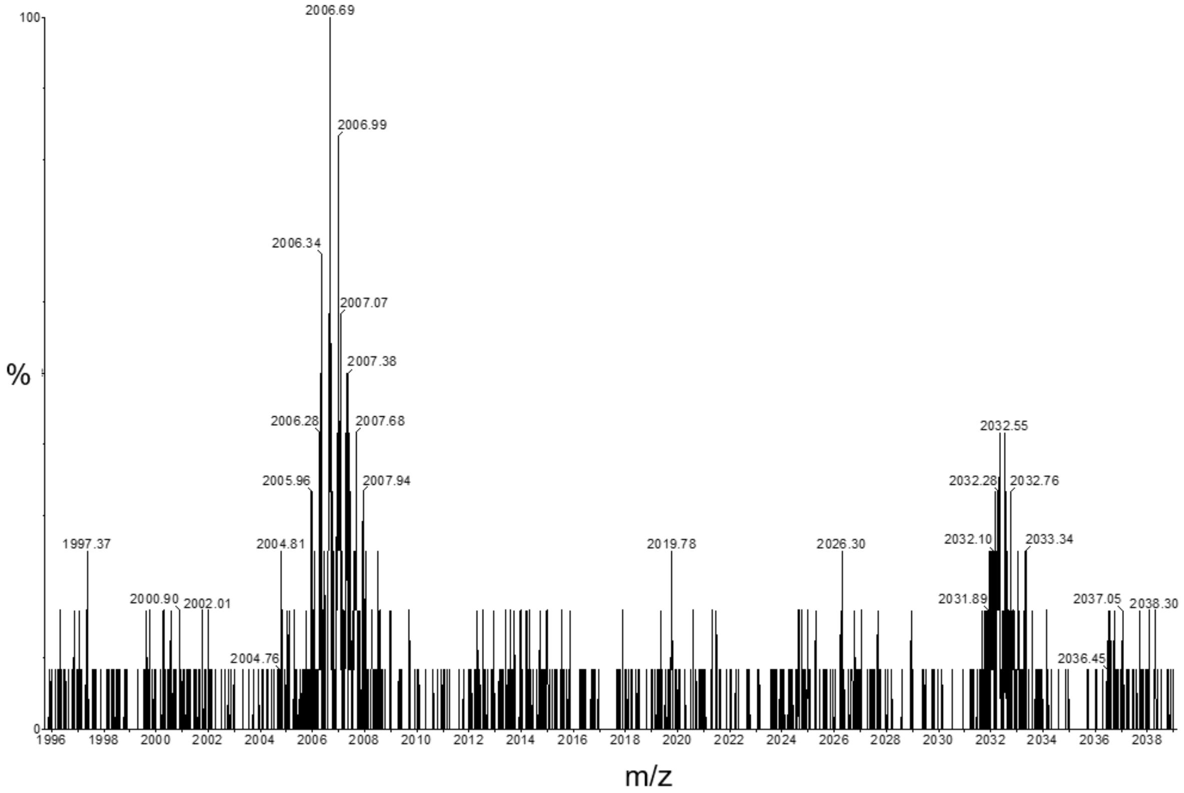

Figure S7. Liquid chromatography – mass spectrometry (LC-MS) of the synthesised FITC-Dextran, acquired using a C4 LC column. The data reveal two triply-charged components with m/z (mass/charge) values of approximately 2007 and 2033 a.m.u. respectively (i.e. molecular weights of 6021 g/mol and 6099 g/mol). These values are consistent with labelling of 6 kDa dextran with FITC. The peak with molecular weight of 6099 g/mol is consistent with a dextran molecule containing 35 units of glucose modified with one FITC molecule and one sodium molecule (which is normally associated to molecules under MS conditions). The peak with molecular weight of 6021 g/mol is consistent with a dextran molecule containing 32 units of glucose modified with two FITC molecules and one potassium molecule (which is normally associated to molecules under MS conditions).

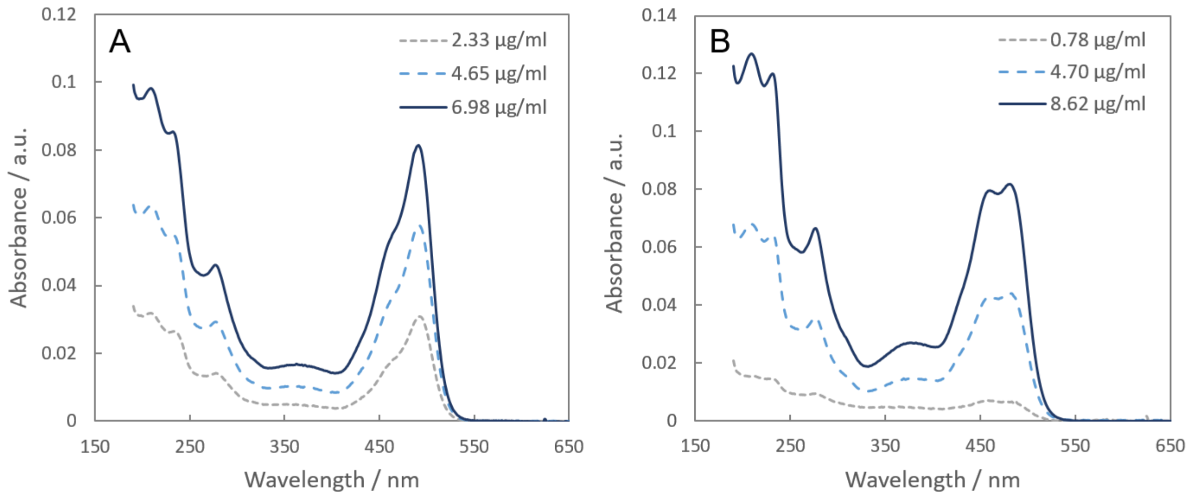

Figure S8. Absorption spectra of synthesised FITC-Dextrans. **(A)** 2.5 eq sample. **(B)** 5 eq sample. Measurements were made at three concentrations to allow calculation of the molar concentrations of FITC. The double-peaked spectral shape observed over the wavelength range 450-500 nm in **(B)** indicated aggregation of FITC molecules in the 5 eq sample.

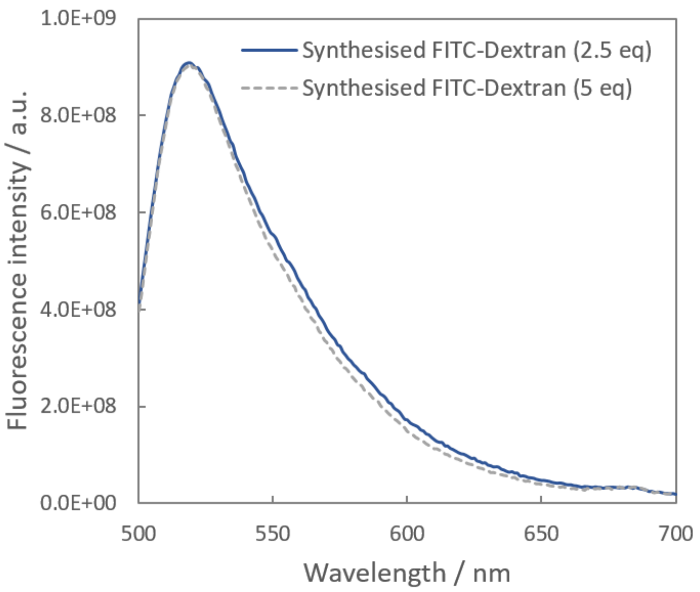

Figure S9. Fluorescence spectra of synthesised FITC-Dextrans. Despite the expected differences in concentration, the 5 eq and 2.5 eq samples exhibited almost identical fluorescence intensities. This was attributed to aggregation and quenching in the 5 eq sample.

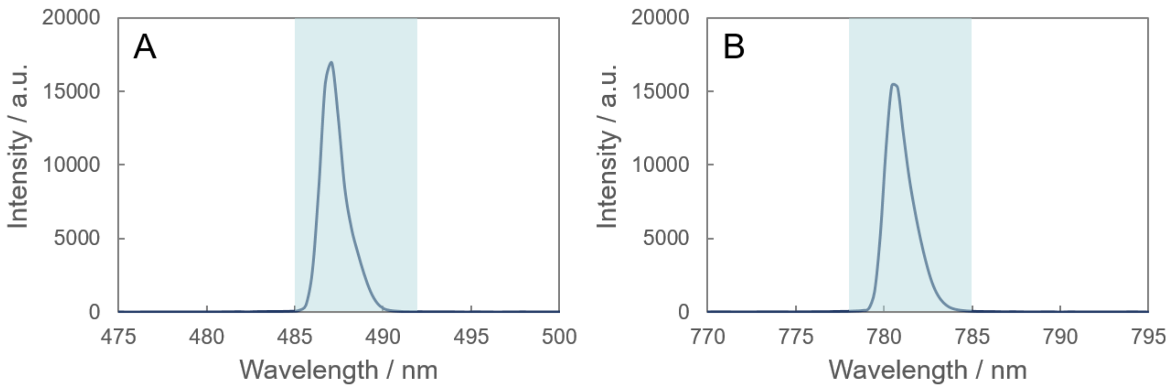

Figure S10. Backscattered laser spectra used for normalisation of fluorescence signals. **(A)** 488 nm laser spectrum. **(B)** 785 nm laser spectrum. Blue shaded regions indicate the spectral ranges summed to obtain the laser power values for normalisation (485-492 nm for the 488 nm laser; 778-785 nm for the 785 nm laser).

Supplementary Table

Table S1. Ingredients of milkshake used in small intestinal absorption experiment (participant 4, experiment vi). Fluorescence intensity was compared against blood concentrations of orally ingested paracetamol (which is known to be absorbed in the small intestine).

Ingredient	Amount
Nesquik Chocolate powder	27 g
Semi-skimmed milk	400 ml
Nutricia PROTIFAR – High Protein Powder	10.4 g
Sugar	2.88 g
Fluorescein	500 mg / 5 ml
Paracetamol	1.5 g

Supplementary Calculations

Mass of FITC in 1 g commercial FITC-Dextran

In the experiment in which commercial FITC-Dextran (FD4, Sigma Aldrich, USA) was consumed (participant 5, experiment iii), the participant received a 1 g oral dose. As the FITC-Dextran had a molecular weight of 4000 g/mol (average molecular weight quoted as 3000–5000 g/mol), the number of moles of FITC-Dextran could be calculated as

$$N_{FITC-Dextran} = {}^{1}/_{4000}\ \text{mol} \tag{S1}$$

Dextran is a polymer of glucose, and in its polymerised form glucose has a molecular weight of 162 g/mol. Thus, the equivalent number of moles of glucose in 1 mole of FITC-Dextran can be approximated as

$$N_{Glucose}(1\ \text{mol}\ FD) = {}^{4000}/_{162} = 24.7\ \text{mol} \tag{S2}$$

Note that the above equation ignores the molecular weight of FITC and the molecular weights of the hydrogen (H) atom and hydroxide (OH) molecule located at each terminus of the dextran chain. This is justified due to the wide molecular weight range quoted by the supplier, which means that the H, OH, and FITC molecular weights would have only a small impact on the calculation.

Based on equation S2, the number of moles of glucose in a 1 g dose of FITC-Dextran can then be calculated as

$$N_{Glucose}(1\ \text{g}\ FD) = {}^{24.7}/_{4000}\ \text{mol} \tag{S3}$$

The supplier (Sigma-Aldrich) quoted the FITC:glucose molar ratio as 0.002–0.02 moles of FITC per mole of glucose. Thus, the number of moles of FITC in a 1 g FITC-Dextran dose could be calculated as

$$N_{FITC}(\text{max}) = 0.02 \times \frac{24.7}{4000}\ \text{mol} \tag{S4}$$

$$N_{FITC}(\text{min}) = 0.002 \times \frac{24.7}{4000}\ \text{mol} \tag{S5}$$

Hence, the mass of FITC (m_{FITC}) in the 1 g dose of FITC-Dextran could then be calculated by multiplying the number of moles of FITC (N_{FITC}) by the molecular weight (MW_{FITC} = 389 g/mol):

$$m_{FITC} = N_{FITC} \times MW_{FITC} \tag{S6}$$

Performing the above calculation for the maximum and minimum values of N_{FITC} (equations S4 and S5) yielded a mass range of **4.8–48 mg FITC** in a 1 g dose of FITC-Dextran.

Molar FITC concentration in synthesized FITC-Dextran (2.5 eq sample)

The molar concentration of FITC (i.e. the FITC:dextran ratio) in the synthesized FITC-Dextran (2.5 eq sample) was calculated based on absorption spectra recorded at three different concentrations (Figure S8A) and the known extinction coefficient of FITC (77,000 $M^{-1}cm^{-1}$ at 494 nm). This calculation was performed as follows.

The extinction coefficient, *ε*, of a substance is related to absorbance, *A*, and concentration, *C*, according to

$$\varepsilon = {}^{A}/_{C} \tag{S7}$$

Thus, the concentration can be written as

$$C = {}^{A}/_{\varepsilon} \tag{S8}$$

For the three FITC-Dextran concentrations, the peak absorbance values at 494 nm were measured as 0.0305, 0.0569 and 0.0796 (for FITC-Dextran concentrations of 2.33 µg/ml, 4.65 µg/ml and 6.98 µg/ml respectively). The concentrations of FITC in each solution can then be calculated using equation S8:

$$C_1 = {}^{A_1}/_{\varepsilon} = {}^{0.0305}/_{77{,}000} = 3.96 \times 10^{-7}\ \mathrm{M} \tag{S9}$$

$$C_2 = {}^{A_2}/_{\varepsilon} = {}^{0.0569}/_{77{,}000} = 7.39 \times 10^{-7}\ \mathrm{M} \tag{S10}$$

$$C_3 = {}^{A_3}/_{\varepsilon} = {}^{0.0796}/_{77{,}000} = 1.03 \times 10^{-6}\ \mathrm{M} \tag{S11}$$

From the above concentrations, the number of moles of FITC, *N*, can be calculated for each solution, based on the volume, *V*, of each sample.

$$N = C \times V \tag{S12}$$

$$N_1 = C_1 \times (2005 \times 10^{-6}) = 7.94 \times 10^{-10}\ \mathrm{mol} \tag{S13}$$

$$N_2 = C_2 \times (2010 \times 10^{-6}) = 1.49 \times 10^{-9}\ \mathrm{mol} \tag{S14}$$

$$N_3 = C_3 \times (2015 \times 10^{-6}) = 2.08 \times 10^{-9}\ \mathrm{mol} \tag{S15}$$

The three solutions at the above concentrations were made up based on a 3 ml stock solution containing 3.79 mg FITC-Dextran. From that stock solution, the three solutions used for absorbance measurements were prepared by diluting 5 µl, 10 µl and 15 µl of stock solution into 2 ml of distilled water (giving total volumes of 2005, 2010 and 2015 µl respectively, as used in equations S13-S15). Hence, the number of moles of FITC in the stock solution can be calculated according to

$$N_{stock} = N \times \frac{V_{total}}{V} \tag{S16}$$

where N_{stock} is the number of moles of FITC in the stock solution, *N* is the number of moles of FITC in each of the three solutions used for absorbance measurements (i.e. the values from equations S13-S15), V_{total} is the total volume of the stock solution (3 ml), and *V* is the volume of stock solution used in each solution for absorbance measurements (i.e. 5 µl, 10 µl or 15 µl). Calculating the value of N_{stock} based on the values from equations S13-S15 and taking the mean value yields

$$N_{stock} = 4.46 \times 10^{-7}\ \mathrm{mol} \tag{S17}$$

The mass of FITC in the 3 ml stock solution, m_{FITC}, can then be calculated as

$$m_{FITC} = N_{stock} \times MW_{FITC} \tag{S18}$$

where MW_{FITC} is the molecular weight of FITC (389 g/mol). Thus,

$$m_{FITC} = (4.46 \times 10^{-7}) \times (389 \times 10^{3}) = 0.17 \text{ mg} \tag{S19}$$

The mass of dextran, m_D, in the sample is then simply given by the difference between the mass of FITC-Dextran in the stock solution (3.79 mg) and the mass of FITC:

$$m_D = m_{FITC-Dextran} - m_{FITC} \tag{S20}$$

$$m_D = 3.79 - 0.17 = 3.62 \text{ mg} \tag{S21}$$

The number of moles of dextran, N_D, can then be calculated according to

$$N_D = {}^{m_D}/_{MW_{Dextran}} \tag{S22}$$

where $MW_{Dextran}$ is the molecular weight of the dextran used for synthesis (6000 g/mol). Hence,

$$N_D = {}^{3.62}/_{(6000 \times 10^{3})} = 6.03 \times 10^{-7} \text{ mol} \tag{S23}$$

As such, the molar ratio of FITC:dextran, $R_{F:D}$, can be calculated as

$$R_{F:D} = \frac{N_{stock}}{N_D} = \frac{4.46\times10^{-7}}{6.03\times10^{-7}} = 0.74 \tag{S24}$$

Multiplying by the molecular weight of glucose in its polymerised form (162 g/mol) and dividing by the molecular weight of dextran (6000 g/mol) then converts this into the FITC:glucose ratio. This was calculated as **0.02 moles of FITC per mole of glucose**.

As discussed in the main text, the FITC:glucose ratio obtained in the synthesised FITC-Dextran is up to 10 times higher than the equivalent molar ratio in the commercial product (0.002–0.02 moles of FITC per mole of glucose). This is consistent with the 3.7-fold increase observed in the fluorescence intensity of the synthesised product relative to the commercial product (Figure 6B).
